# Analytical solution for bending vibration of a thin-walled cylinder rolling on a time-varying force

**DOI:** 10.1098/rsos.180639

**Published:** 2018-07-11

**Authors:** A. Le Bot, G. Duval, P. Klein, J. Lelong

**Affiliations:** 1 Université de Lyon, Lyon, France; 2 CNRS, Laboratoire de Tribologie et Dynamique des Systèmes, Ecole centrale de Lyon, Ecully, France; 3 IFSTTAR, Laboratoire d'acoustique environnementale, Bron, France

**Keywords:** sound and vibration, thin shells, moving force, rolling cylinder, tyre–road noise

## Abstract

This paper presents the analytical solution of radial vibration of a rolling cylinder submitted to a time-varying point force. In the simplest situation of simply supported edges and zero in-plane vibration, the cylinder is equivalent to an orthotropic pre-stressed plate resting on a visco-elastic foundation. We give the closed-form solution of vibration as a series of normal modes whose coefficients are explicitly calculated. Cases of both deterministic and random forces are examined. We analyse the effect of rolling speed on merging of vibrational energy induced by Doppler's effect for the example of rolling tyre.

## 1. Introduction

Rolling of thin-walled cylinders on rough surfaces generates vibration responsible for sound radiation. This situation is encountered in many engineering problems like noise of tyre/ road [1] or rail/wheel contact [2].

The canonical problem associated with these situations is that of a time-varying point force moving in the circumferential direction of the cylinder with a constant rotational speed. Since the frame attached to the cylinder is not Galilean, there are Coriolis and centrifugal forces. In particular, the gyroscopic effect breaks the symmetry between onward and backward circumferential waves. This results in a bifurcation which separates eigenfrequencies initially degenerated for zero rolling speed [3,4].

Various solutions to this problem have been proposed in the literature. The cases of constant and harmonic forces may be found in [5,6]. Owing to the complexity of cylindrical shell equations, these solutions rely on a numerical determination of mode constants.

In this study, we shall show that in the simplest situation of simply supported edges and zero in-plane deformation, it is possible to completely solve the problem of a time-varying force turning on a cylinder even in the presence of bending and membrane orthotropy and random force.

## 2. Rotating cylindrical shell

**Figure 1.**
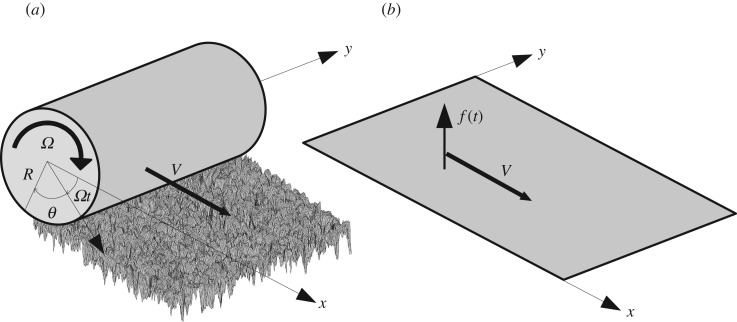
(*a*) Cylinder rolling on a rough surface. (*b*) Equivalent infinite flat periodic plate with periodic force field moving in *x*-direction.

Consider a cylinder of radius *R* rolling on a rough surface with rotational speed *Ω* and translational speed *V* = *ΩR* as shown in figure 1*a*. We denote by *y* the axial position and *θ* the angular position of a point in the frame rotating with the cylinder.

The cylinder is a thin-walled shell and is deformed by contact forces while nominally flat and the surface is rigid. For rough surfaces, the contact is rare and confined into few contact spots of small area and randomly distributed [7]. The wavelength of vibration being generally much larger than the typical size of contact spots, the contact forces may be, therefore, idealized by point random forces.

There exists a wide variety of theories for thin-walled cylindrical shells in elastic deformation. Among these, we shall choose the linear theory considering small deformation **u** = (*u*, *v*, *w*), where *u* is the axial deflection, *v* the circumferential deflection and *w* the radial deflection. Since the external forces are radial, we shall also focus on the radial deflection *w*(*y*, *θ*, *t*). A popular theory introduced by Lord Rayleigh is the bending approximation also known as inextensional approximation. It consists of assuming that in-plane deformations *ϵ*_*yy*_, *ϵ*_*yθ*_, *ϵ*_*θθ*_ are zero and leads to the governing equation

2.1 $$m\frac{\partial^{2}w}{\partial t^{2}}+D{\left(\frac{\partial^{2}}{\partial y^{2}}+\frac{1}{R^{2}}\frac{\partial^{2}}{\partial \theta^{2}}\right)}^{2}w-\frac{D}{R^{2}}\left(\frac{\partial^{2}}{\partial y^{2}}+\frac{1}{R^{2}}\frac{\partial^{2}}{\partial \theta^{2}}\right)w=0,$$

where *D* = *Eh*^3^/12(1 − *ν*^2^) is the bending rigidity, *E* Young's modulus, *h* the shell thickness, *ν* Poisson's coefficient and *m* the mass per unit area. The second term is the bi-Laplacian operator associated with bending while the third term is the Laplacian operator for membrane effect. An alternative theory for out-of-plane motion of shells is obtained by assuming that in-plane deflections *u*, *v* are zero. In that case (see [8, eqn (6.5.7), p. 153])

2.2 $$m\frac{\partial^{2}w}{\partial t^{2}}+D{\left(\frac{\partial^{2}}{\partial y^{2}}+\frac{1}{R^{2}}\frac{\partial^{2}}{\partial \theta^{2}}\right)}^{2}w+\frac{K}{R^{2}}w=0,$$

where *K* = *Eh*/(1 − *ν*^2^) is the membrane stiffness. In the next sections, we shall solve an equation that generalizes both (2.1) and (2.2). Other theories, such as Donnell–Mushtari–Vlasov, result in the appearance of higher order spatial derivatives.

Since the cylinder rotates, we must modify the governing equations and include the effect of inertial forces. See for instance [9] for a complete treatment with Donnell–Mushtari–Vlasov equations. In the rotating frame, the movement of a point is first affected by the Coriolis force $-2m\Omega y\times \dot{u}$ whose radial contribution is $2m\Omega \dot{v}$. Under the zero in-plane deflection approximation, *v* = 0 and the Coriolis force vanishes.

The radial centrifugal force *mΩ*^2^(*R* + *w*) is a sum of static contribution and a dynamic term. The dynamic term *mΩ*^2^*w* is an elastic force but with negative stiffness. The contribution to the governing equation is, therefore,

2.3 $$-m\Omega^{2}w.$$

The static term *mΩ*^2^*R* contributes to increase by *mΩ*^2^*R*^2^ the membrane tension in the circumferential direction. The contribution to the governing equation is, therefore, a term like *mΩ*^2^∂^2^_*θθ*_*w*. More generally, if the cylinder is filled with a gas of pressure *p*, the contribution to the governing equation is

2.4 $$-pR\frac{\partial^{2}w}{\partial y^{2}}-(pR+m\Omega^{2}R^{2})\frac{1}{R^{2}}\frac{\partial^{2}w}{\partial \theta^{2}}.$$

The *y*- and *θ*-derivative being multiplied by different coefficients (due to the presence of *mΩ*^2^*R*^2^ in the second term), this does not reduce to the Laplacian. The membrane operator of the rotating cylindrical shell is, therefore, anisotropic.

If we unwrap the cylinder, we obtain an equivalent thin plate with periodic boundary conditions in the longitudinal direction excited by point forces. By linearity, it suffices to solve the problem for a unique force and then to apply linear superposition in the case of several forces. Furthermore, the rotation of the cylinder leads to a circumferential movement of contact and, therefore, the point force is moving in the longitudinal direction with speed *V* in the equivalent infinite periodic plate like in figure 1*b*.

We shall, therefore, solve the problem of a periodic thin plate excited by a moving point force of random nature. For practical purposes, we shall include bending anisotropy in our plate model. This picture will represent a good approximation of the previously mentioned problem of a rolling cylinder as soon as the radial vibration dominates all other movements.

## 3. Boundary-value problem

The governing equation for out-of-plane motion *w*(*x*, *y*, *t*) of a vibrating plate is

3.1 $$m\frac{\partial^{2}w}{\partial t^{2}}+c\frac{\partial w}{\partial t}+Kw=f(x,y,t),$$

where *m* is the mass per unit area, *c* is the viscous damping coefficient and K the stiffness operator. The choice of linear damping will be important later for developing the solution in normal mode series. The most general form of the operator K is

3.2 $$Kw=\left(B_{x}\frac{\partial^{4}}{\partial x^{4}}+2\sqrt{B_{x}B_{y}}\frac{\partial^{4}}{\partial x^{2}y^{2}}+B_{y}\frac{\partial^{4}}{\partial y^{4}}\right)w-\left(T_{x}\frac{\partial^{2}}{\partial x^{2}}+T_{y}\frac{\partial^{2}}{\partial y^{2}}\right)w+Sw.$$

This operator contains three terms. The derivatives in the first parentheses constitute the thin orthotropic plate operator for flexural motion where *B*_*x*_, *B*_*y*_ are the bending stiffnesses. This is a fourth-order operator which reduces to bi-Laplacian Δ^2^. = (∂^2^/∂*x*^2^ + ∂^2^/∂*y*^2^)^2^ for isotropic plates (*B*_*x*_ = *B*_*y*_). The second parentheses contain the membrane operator. The tension *T*_*x*_ and *T*_*y*_ may include the effects of curvature, inflation pressure and centrifugal force of the rolling cylinder. In the case of equal tensions (*T*_*x*_ = *T*_*y*_), this operator reduces to Laplacian Δ. = ∂^2^/∂*x*^2^ + ∂^2^/∂*y*^2^. The last term *Sw* is a restoring force (Winkler foundation), where *S* include the dynamic centrifugal force and effect of curvature. Let us remark that this general form of the operator K includes equations (2.1) and (2.2) as special cases.

Since the stiffness operator K is of order 4, we must specify two boundary conditions per edge. First, the plate is simply supported on its lateral edges. Since the plate has width *b*, the deflection *w* is zero at *y* = 0 and at *y* = *b*. Then

3.3 0=w(x,0,t)

and

3.4 0=w(x,b,t).

The second condition imposed by a simply supported edge is a null bending moment that is ∂_*y*^2^_*w* + *ν*∂_*x*^2^_*w* = 0. But ∂_*x*^2^_*w* = 0 on the edge and, therefore, the two conditions become

3.5 $$0=\frac{\partial^{2}w}{\partial y^{2}}(x,0,t)$$

and

3.6 $$0=\frac{\partial^{2}w}{\partial y^{2}}(x,b,t).$$

Note that the Poisson ratio *ν* no longer appears.

In the longitudinal direction, the plate is infinite but periodic with period *a* = 2*πR*. The unique condition is, therefore,

3.7 w(x,y,t)=w(x+a,y,t),

for all *x* and *y*. Let us remark that periodicity of *w* implies those of all derivatives ∂_*x*^*m*^*y*^*n*^_*w* of any order such that rotation of cross section, bending moment and shear force are also periodic functions.

The boundary-value problem is constituted by equation (3.1), boundary conditions (3.3)–(3.6) and periodic condition (3.7).

## 4. Green's function

The normal modes are defined as a sequence of eigenvectors of the stiffness operator K and submitted to the boundary conditions (3.3)–(3.7). These are functions of *x*, *y* and they verify Kψ=λψ, where λ is the related eigenvalue. Conventionally, we assumed that the eigenmodes are normalized by $\int \int \psi {\left(x,y\right)}^{2}\;dx\;dy=1$.

Eigenvalues and eigenmodes are found by the classical method of separation of variables. Setting *ψ*(*x*, *y*) = *f*(*x*)*g*(*y*), we obtain two ordinary differential equations on *f* and *g* that may be solved separately. We find two types of eigenmodes

4.1 $$\begin{array}{rl} \psi_{i,j,1}(x,y) & =\sqrt{\frac{4\epsilon_{i}}{ab}}\cos\left(2i\pi \frac{x}{a}\right)\sin\left(j\pi \frac{y}{b}\right)\\ \;\mathrm{and}\;\psi_{i,j,2}(x,y) & =\sqrt{\frac{4\epsilon_{i}}{ab}}\sin\left(2i\pi \frac{x}{a}\right)\sin\left(j\pi \frac{y}{b}\right), \end{array}\}$$

where *ϵ*_0_ = 1/2, *ϵ*_*i*_ = 1 if *i*≠0 and *i*≥0, *j*≥1 are two integers. The constant 4*ϵ*_*i*_/*ab* is chosen to satisfy the normalization condition. The two modes (4.1) have the same eigenvalue λ_*ij*_ given by

4.2 $$\lambda_{ij}=m\omega_{ij}^{2}={\left[B_{x}^{1/2}{\left(\frac{2i\pi }{a}\right)}^{2}+B_{y}^{1/2}{\left(\frac{j\pi }{b}\right)}^{2}\right]}^{2}+T_{x}{\left(\frac{2i\pi }{a}\right)}^{2}+T_{y}{\left(\frac{j\pi }{b}\right)}^{2}+S.$$

When *i*≠0, the two modes (4.1) are linearly independent and therefore the eigenspace has dimension 2. But when *i* = 0, the second mode (4.1) degenerates to zero and therefore the eigenspace has dimension 1.

In what follows, we shall introduce the modal damping factor *ζ*_*ij*_ = *c*/2 *mω*_*ij*_ and the reduced circular frequency *ω*′_*ij*_ = *ω*_*ij*_(1 − *ζ*^2^_*ij*_)^1/2^. We, therefore, restrict the study to the small damping case that is *ζ*_*ij*_ < 1 for all *i*≥0 and *j*≥1.

We denote by *h*(*x*, *y*, *x*′, *y*′;*t*) the Green function, or impulse response, of the plate. The observation point, or receiver, is at position (*x*, *y*) while (*x*′, *y*′) is the position of where a shock of unit impulse is applied at time *t* = 0. The Green function is solution to

4.3 $$\begin{array}{rl} & m\frac{\partial^{2}h}{\partial t^{2}}+c\frac{\partial h}{\partial t}+Kh=\delta (t)\delta (x-x^{\prime })\delta (y-y^{\prime })\\ & \;\text{boundary conditions (3.3)–(3.7)}\\ \;\mathrm{and}\; & h(x,y,t)=\frac{\partial h}{\partial t}(x,y,t)=0\;\text{for}\;t\leq 0. \end{array}\}$$

Since we have assumed a damping force of viscous type *c*∂_*t*_*w* with a ratio *c*/*m* that does not depend on position, we are allowed to find the solution to problem (4.3) by an expansion as a normal mode series $h=\sum_{\alpha }A_{\alpha }(t)\psi_{\alpha }(x,y)$ where the sum runs over all *α* = (*i*, *j*, *k*). Remarking that such a decomposition naturally verifies the boundary conditions and substituting this form into the first equation (4.3) give

4.4 $$h(x,y,x^{\prime },y^{\prime };t)=\sum_{i,j,k}h_{ij}(t)\psi_{ijk}(x,y)\psi_{ijk}(x^{\prime },y^{\prime }),$$

where

4.5 $$h_{ij}(t)=\frac{H(t)}{m\omega_{ij}^{\prime }}e^{-\zeta_{ij}\omega_{ij}t}\sin\omega_{ij}^{\prime }t$$

and *H*(*t*) = 0 if *t* < 0 and *H*(*t*) = 1 otherwise. By expanding with the eigenmodes (4.1) and simplifying, it yields [10]

4.6 $$h(x,y,x^{\prime },y^{\prime };t)=\sum_{i=0}^{\infty }\sum_{j=1}^{\infty }\frac{4\epsilon_{i}}{ab}h_{ij}(t)\cos\left(2i\pi \frac{x-x^{\prime }}{a}\right)\sin\left(j\pi \frac{y}{b}\right)\sin\left(j\pi \frac{y^{\prime }}{b}\right),$$

where $\epsilon_{0}=\frac{1}{2}$ and *ϵ*_*i*_ = 1 if *i*≠0. This is the Green function of an orthotropic pre-stressed plate on visco-elastic foundation with longitudinal periodic conditions and lateral simply supported edges. Let us remark that the Green function is time-invariant (*h* does not depend on the time at which the impulse is applied but just on the time delay) and causal (*h* = 0 when *t* ≤ 0). Furthermore, *h* does not depend on separately *x* and *x*′ but only on their difference *x* − *x*′.

It may also be of interest to calculate the time-derivative of the Green function denoted ∂_*t*_*h*. This function ∂_*t*_*h* provides information on the vibrational velocity while *h* gives the vibrational displacement. First, we differentiate (4.5):

4.7 $${\dot{h}}_{ij}(t)=\frac{H(t)}{m\omega_{ij}^{\prime }}e^{-\zeta_{ij}\omega_{ij}t}[-\zeta_{ij}\omega_{ij}\sin\omega_{ij}^{\prime }t+\omega_{ij}^{\prime }\cos\omega_{ij}^{\prime }t]$$

(we may remark that the time-derivative of the Heaviside function *H*(*t*) does not contribute to the result since it is multiplied by the function sin(*ω*′_*ij*_*t*) which is null at *t* = 0). We get

4.8 $$\frac{\partial h}{\partial t}(x,y,x^{\prime },y^{\prime };t)=\sum_{i=0}^{\infty }\sum_{j=1}^{\infty }\frac{4\epsilon_{i}}{ab}{\dot{h}}_{ij}(t)\cos\left(2i\pi \frac{x-x^{\prime }}{a}\right)\sin\left(j\pi \frac{y}{b}\right)\sin\left(j\pi \frac{y^{\prime }}{b}\right).$$

The two equations (4.6) and (4.8) will be useful for derive the solution of (3.1) for a moving point force.

## 5. Moving force solution

We now consider a time-varying point force moving in the *x*-direction with a constant velocity *V* . The boundary-value problem with initial conditions to solve is

5.1 $$\begin{array}{rl} & m\frac{\partial^{2}w}{\partial t^{2}}+c\frac{\partial w}{\partial t}+Kw=f(t)\delta (x-Vt)\delta (y-y_{0})\\ & \text{boundary conditions (3.3)–(3.7)}\\ \;\mathrm{and}\; & \lim_{t\to -\infty }w(x,y,t)=\lim_{t\to -\infty }\frac{\partial w}{\partial t}(x,y,t)=0, \end{array}\}$$

where *δ* denotes the Dirac function. The point force position at time *t* is *x* = *V* *t* and *y* = *y*_0_. The time history of the force *f*(*t*) may be arbitrary. The initial conditions when *t* →  − ∞ are chosen such that the plate is initially at rest.

The solution to problem (5.1) is obtained by a convolution of the force field with the Green function [11]

5.2 $$\begin{array}{rl} w(x,y,t) & =\int_{0}^{+\infty }d\tau \int_{0}^{a}dx^{\prime }\int_{0}^{b}dy^{\prime }h(x,y,x^{\prime },y^{\prime };\tau )f(t-\tau )\delta (x^{\prime }-V(t-\tau ))\delta (y^{\prime }-y_{0}) \end{array}$$

5.3 $$\begin{array}{rl} & =\int_{-\infty }^{\infty }h(x,y,V(t-\tau ),y_{0};\tau )f(t-\tau )\;d\tau , \end{array}$$

where the lower bound of the integral has been extended to −∞ since *h*(*x*, *y*, *x*′, *y*′;*τ*) = 0 if *τ* < 0.

Let us fix the position of the observation point at a constant distance to the point force. The observation point is, therefore, moving at speed *V* relative to the plate. We must, therefore, substitute *x* → *x* + *V* *t* in equation (5.3)

5.4 $$w(x+Vt,y,t)=\int_{-\infty }^{\infty }h(x+Vt,y,V(t-\tau ),y_{0};\tau )f(t-\tau )\;d\tau .$$

But *h*(*x* + *V* *t*, *y*, *V* (*t* − *τ*), *y*_0_;*τ*) = *h*(*x* + *V* *τ*, *y*, 0, *y*_0_;*τ*) by virtue of a previous remark. Then the solution becomes

5.5 $$w(x+Vt,y,t)=\int_{-\infty }^{\infty }h(x+V\tau ,y,0,y_{0};\tau )f(t-\tau )\;d\tau .$$

The solution is therefore a convolution product between the force *τ*↦*f*(*τ*) and the kernel *τ*↦*h*(*x* + *V* *τ*, *y*, 0, *y*_0_;*τ*).

It is immediate to observe that the vibrational velocity ∂_*t*_*w* at the moving receiver *x* + *V* *t*, *y* is also obtained by a convolution product with the force. The calculation follows in the same way from equations (5.2) to (5.5) but with ∂_*t*_*h* in place of *h*. The result is

5.6 $$\frac{\partial w}{\partial t}(x+Vt,y,t)=\int_{-\infty }^{\infty }\frac{\partial h}{\partial t}(x+V\tau ,y,0,y_{0};\tau )f(t-\tau )\;d\tau ,$$

where the kernel is now *τ*↦∂_*t*_*h*(*x* + *V* *τ*, *y*, 0, *y*_0_;*τ*) given in equation (4.8). It must be noticed that the vibrational velocity ∂_*t*_*w* is that of a point fixed in the frame attached to the plate, and in the case of a rotating cylinder, that of a point fixed in the rotating frame. Since this is the velocity of a particle of matter in the frame where its mean velocity is null, ∂_*t*_*w* is called Lagrangian derivative in the following. But it may be also of interest especially for the purpose of sound radiation to estimate the vibrational velocity d_*t*_*w* of a point fixed in the frame moving with the force, called Eulerian velocity. This gives

5.7 $$\frac{dw}{dt}(x+Vt,y,t)=\int_{-\infty }^{\infty }h(x+V\tau ,y,0,y_{0};\tau )\dot{f}(t-\tau )\;d\tau ,$$

which must be understood as the time-derivative of *t*↦*w*(*x* + *V* *t*, *y*, *t*) obtained from equation (5.5).

We now restrict to the case of a harmonic force. By substituting *f*(*t*) = e^ı*ωt*^ into equation (5.5), we observe that the solution is of the form *w*(*x* + *V* *t*, *y*, *t*) = *H*(*x*, *y*, *y*_0_;*ω*)e^ı*ωt*^, where

5.8 $$H(x,y,y_{0};\omega )=\int_{-\infty }^{\infty }h(x+V\tau ,y,0,y_{0};\tau )e^{-ı\omega \tau }\;d\tau$$

is the frequency response function between the moving force at position (*V* *t*, *y*_0_) and vibrational displacement at position (*x* + *V* *t*, *y*). In the following, this function is called receptance of the rolling cylinder.

By substituting the Green function (4.6) into equation (5.8), it yields

5.9 $$H(x,y,y_{0};\omega )=\sum_{i=0}^{\infty }\sum_{j=1}^{\infty }\frac{4\epsilon_{i}}{mab\omega_{ij}^{\prime }}I_{ij}(x,\omega )\sin\left(j\pi \frac{y}{b}\right)\sin\left(j\pi \frac{y_{0}}{b}\right),$$

where

5.10 $$I_{ij}(x,\omega )=\int_{0}^{\infty }e^{-\zeta_{ij}\omega_{ij}\tau }\sin\omega_{ij}^{\prime }\tau \cos\left(2i\pi \frac{x+V\tau }{a}\right)e^{-ı\omega \tau }\;d\tau .$$

This integral is calculated in equation (A 11).

Concerning the vibrational velocity, substituting *f*(*t*) = e^ı*ωt*^ into equation (5.5) also gives a solution of the form ∂_*t*_*w*(*x* + *V* *t*, *y*, *t*) = *Y* (*x*, *y*, *y*_0_;*ω*)e^ı*ωt*^, where *Y* is the frequency response function between the moving force and the Lagrangian vibrational velocity at receiver. This is the mobility of the rolling cylinder

5.11 $$Y(x,y,y_{0};\omega )=\sum_{i=0}^{\infty }\sum_{j=1}^{\infty }\frac{4\epsilon_{i}}{mab\omega_{ij}^{\prime }}[-\zeta_{ij}\omega_{ij}I_{ij}(x,\omega )+\omega_{ij}^{\prime }J_{ij}(x,\omega )]\sin\left(j\pi \frac{y}{b}\right)\sin\left(j\pi \frac{y_{0}}{b}\right),$$

where

5.12 $$J_{ij}(x,\omega )=\int_{0}^{\infty }e^{-\zeta_{ij}\omega_{ij}\tau }\cos\omega_{ij}^{\prime }\tau \cos\left(2i\pi \frac{x+V\tau }{a}\right)e^{-ı\omega \tau }\;d\tau .$$

This integral is also calculated in equation (A 12). The estimation of the Eulerian speed d_*t*_*w* is even simpler. We find d_*t*_*w*(*x* + *V* *t*, *y*, *t*) = ı*ωH*(*x*, *y*, *y*_0_;*ω*)e^ı*ωt*^ such that ı*ωH* is the apparent mobility estimated in the frame moving with the force.

We now turn to the case of a random force. If *f*(*t*) is a stationary white noise with zero mean and flat spectrum of power *S*_0_ then *w*(*x* + *V* *t*, *y*, *t*) given in equation (5.5) is also a stationary random function with zero mean. The reason is that with the observation point moving at same speed as the force point, the system is time-invariant. This is seen from equation (5.5) which shows that the transformation is linear and the impulse response *τ*↦*h*(*x* + *V* *τ*, *y*, 0, *y*_0_;*τ*) is causal (null for *τ* < 0) and time-invariant (no dependence on time at which the impulse is applied). Then, the theory of linear time-invariant systems gives the power spectral density *S*_*w*_(*ω*) = |*H*(*x*, *y*, *y*_0_;*ω*)|^2^*S*_0_ [12]. So, if the force power spectral density *S*_0_ is confined into the frequency band Δ*ω*, then the probabilistic expectation of the square of *w* is

5.13 $$\langle w^{2}\rangle =\frac{S_{0}}{\pi }\int_{\Delta \omega }|H(x,y,y_{0};\omega ){|}^{2}\;d\omega .$$

Similarly, the probabilistic expectation of the square of Lagrangian vibrational speed at receiver is

5.14 $$\langle \partial_{t}w^{2}\rangle =\frac{S_{0}}{\pi }\int_{\Delta \omega }|Y(x,y,y_{0};\omega ){|}^{2}\;d\omega .$$

Finally, the expectation of the square of Eulerian vibrational speed is

5.15 $$\langle d_{t}w^{2}\rangle =\frac{S_{0}}{\pi }\int_{\Delta \omega }\omega^{2}|H(x,y,y_{0};\omega ){|}^{2}\;d\omega .$$

The vibrational energy density is estimated by taking twice the mean kinetic energy. We may, therefore, distinguish the Lagrangian vibrational energy *E*_∂_ = *m*〈∂_*t*_*w*^2^〉 of a point fixed to the plate and the Eulerian vibrational energy *E*_d_ = *m*〈d_*t*_*w*^2^〉 of a point moving with the force. In the next section, we shall observe maps (*x*, *y*)↦*E*_∂_, *E*_d_ for various speeds *V* and frequency *ω*.

## 6. Application to rolling tyres

In this section, the above described modelling is applied to the case of a rolling tyre. A more elaborate shell model for tyres may be found for instance in [13]. The contact between a tyre and road is confined into a patch of a few centimetres in length and width and is composed of numerous spots of a few millimetres mainly imposed by the size of aggregate of road. The time evolution of contact forces, related to the vehicle speed, presents a wide spectrum up to several kilohertz [14].

Typical values of mechanical parameters for the plate model considered obtained on a slick tyre of size 155/70/R13 are given in [15] and reported in table 1. Geometrical parameters *a* and *b* have been directly measured and correspond, respectively, to the tyre circumference and the sum of tyre belt width and twice the height of side walls. The mass per unit area *m* and the mechanical parameters *B*_*x*_, *B*_*y*_, *T*_*x*_, *T*_*y*_, *S* together with associated damping factors have been identified by curve fitting between theoretical and measured mobility using a simplex search method. Internal losses in the tyre structure materials have also been estimated in [15] by a modal analysis. Typical values of *ζ* are about 2–8% and increase with frequency. In the following simulations, we have chosen *c* = 2*ζωm* = 881 N s m^−3^ (*ζ* = 2.8%) at 200 Hz and *c* = 22 552 N s m^−3^ (*ζ* = 7.2%) at 2 kHz.

**Table 1.** Mechanical parameters of a tyre: *a* = 2*πR* length, *b* width, *m* mass per unit area, *B*_*x*_, *B*_*y*_ bending stiffness, *T*_*x*_, *T*_*y*_ tension, *S* foundation stiffness per unit area.

*a* (m)	*b* (m)	*m* (kg m^−2^)	*B*_*x*_ (N m)	*B*_*y*_ (N m)	*T*_*x*_ (N m^−1^)	*T*_*y*_ (N m^−1^)	*S* (N m^−3^)
1.78	0.32	12.4	20	8	3.0 × 10^4^	8.0 × 10^4^	1.3 × 10^6^

**Figure 2.**
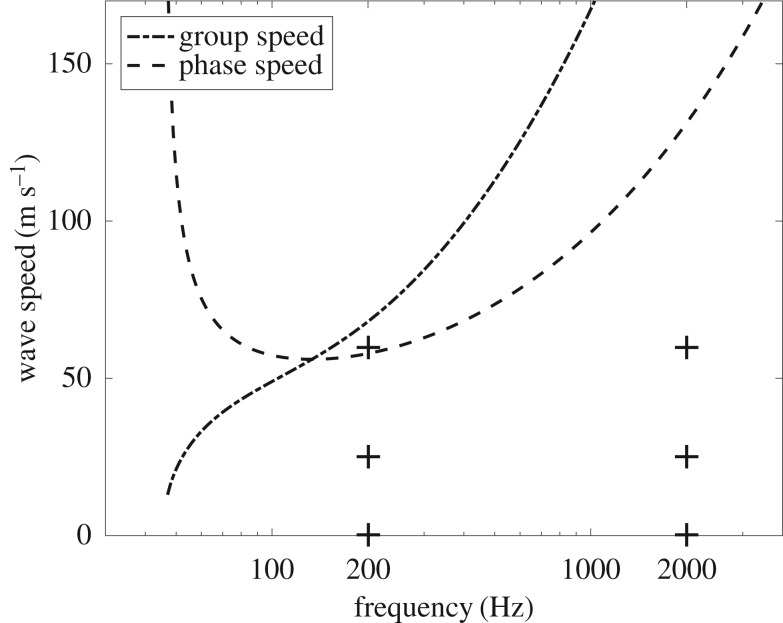
Circumferential phase speed and group speed in tyre versus frequency (logarithmic scale). The crosses indicate the frequency (200 and 2000 Hz) and moving speeds (*V* = 0 km h^−1^, *V* = 90 km h^−1^ and *V* = 216 km h^−1^) of figures 3–6.

As a highly dispersive medium the phase speed *c*_p_ and group speed *c*_g_ strongly depend on frequency. The phase and group speeds in the circumferential direction for the parameters given above are drawn in figure 2 (see [16] for details). The cut-on frequency is 45 Hz. The group speed increases with frequency while the phase speed is infinite just above the cut-on frequency, reaches a minimum between 100 Hz and 200 Hz and then increases with the group speed such that *c*_p_ = *c*_g_/2 (similar to simple plate behaviour). The quite low phase speed of waves is to be noticed. It is especially lower than 100 m s^−1^ between 50 Hz and 1 kHz which makes the rolling speed in typical driving situations not negligible (usually of the order of 30 m s^−1^, for instance, on suburban roads).

Maps of Lagrangian vibrational energy density *E*_∂_ and Eulerian vibrational energy density *E*_d_ have been evaluated for several speeds *V* and frequency bands Δ*ω* according to equations (5.14) and (5.15). The method of calculation follows the same procedure as in [17]. The sum over indices *i* and *j* for the calculation of the cylinder mobility *Y* by equation (5.11) cannot be infinite. Selected modes at frequency *ω* are those for which *ω*_0,*j*_ ≤ 2*ω* and *ω*_*i*,1_ ≤ 2*ω*. The plate is discretized in rectangular elements of size 3.6 mm in the circumferential direction and 3.2 mm in the lateral direction ensuring a fine enough resolution to capture relevant details up to at least 5 kHz. The frequency integration in equations (5.14) and (5.15) is performed over Δ*ω* chosen as one-third octave bands. The frequency domain is subdivided into integration elements twice as many as natural frequencies in Δ*ω*.

**Figure 3.**
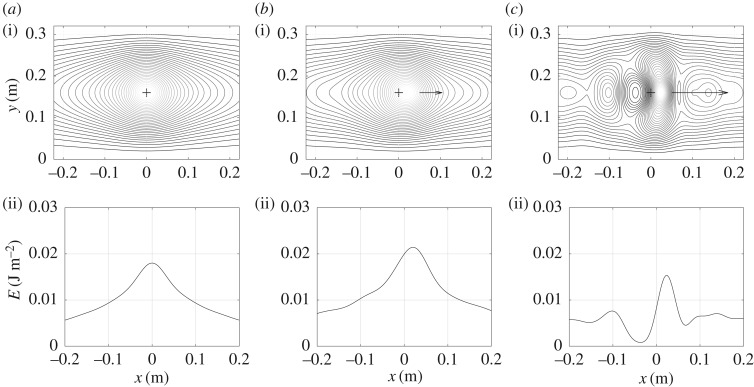
Repartition of Lagrangian vibrational energy *E*_∂_ near the moving point force at *f* = 200 Hz for various moving speeds. Isovalues of energy (i) and energy versus position along the horizontal line *y* = 0.16 (ii): (*a*) *V* = 0 km h^−1^, (*b*) *V* = 90 km h^−1^ and (*c*) *V* = 216 km h^−1^.

**Figure 4.**
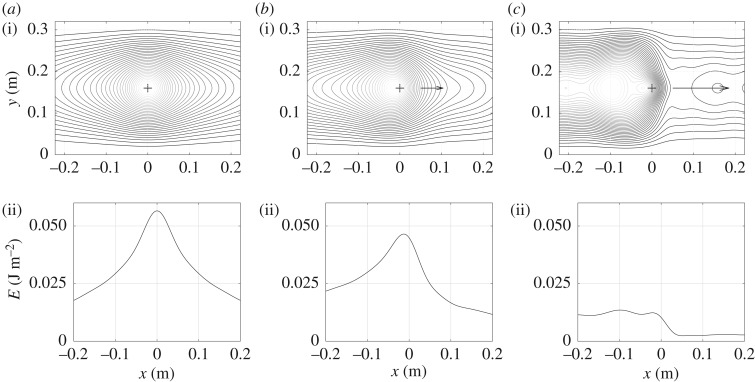
Repartition of Eulerian vibrational energy *E*_d_ near the moving point force at *f* = 200 Hz for various moving speeds. Isovalues of energy (i) and energy versus position along the horizontal line *y* = 0.16 (ii): (*a*) *V* = 0 km h^−1^, (*b*) *V* = 90 km h^−1^ and (*c*) *V* = 216 km h^−1^.

**Figure 5.**
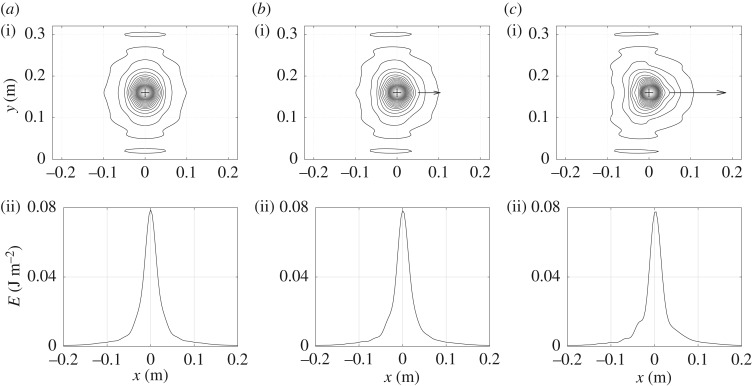
Repartition of Lagrangian vibrational energy *E*_∂_ near the moving point force at *f* = 2000 Hz for various moving speeds. Isovalues of energy (i) and energy versus position along the horizontal line *y* = 0.16 (ii): (*a*) *V* = 0 km h^−1^, (*b*) *V* = 90 km h^−1^ and (*c*) *V* = 216 km h^−1^.

**Figure 6.**
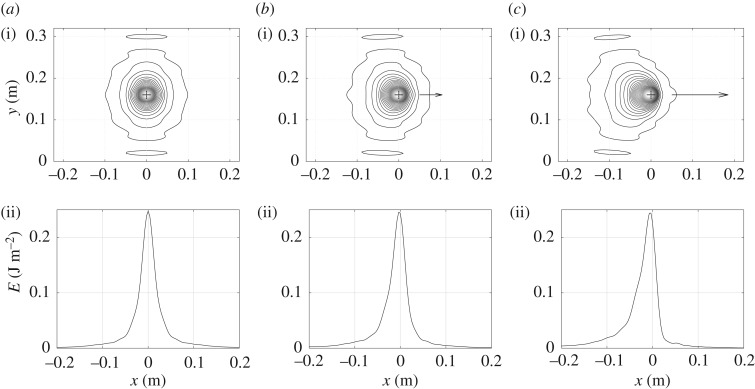
Repartition of Eulerian vibrational energy *E*_d_ near the moving point force at *f* = 2000 Hz for various moving speeds. Isovalues of energy (i) and energy versus position along the horizontal line *y* = 0.16 (ii). (*a*) *V* = 0 km h^−1^, (*b*) *V* = 90 km h^−1^ and (*c*) *V* = 216 km h^−1^.

Two examples of vibrational energy maps are shown in figures 3–6 as contour lines around the excitation and its corresponding evolution as a function of position along medial axis of the tyre. They show typical results obtained over the frequency domain considered. Both give the vibrational energy evaluated for three moving random force velocities *V* = 0 km h^−1^, *V* = 90 km h^−1^ and *V* = 216 km h^−1^. Markers superimposed in figure 2 give their relative position with respect to group and phase speeds.

Figures 3 and 4 concern the one-third octave band 200 Hz characterized by a modal field regime [16]. The phase speed is *c*_p_ = 58 m s^−1^ and the group speed *c*_g_ = 68 m s^−1^. As observed in the maps the attenuation in the lateral and circumferential directions is low. As expected for *V* = 0, the energy is distributed symmetrically with respect to the excitation point (represented by the cross in the graphs). For *V* > 0, the distribution becomes asymmetric due to Doppler's effect. The maximum of energy is moved to the rear of the excitation point. In the vicinity (a few centimetres) of this maximum the energy in the force travelling direction is concentrated. The higher the velocity, the more concentrated the energy. In the opposite direction the reverse occurs. The energy is driven to the rear. It is also to be noticed that at this frequency the maximum energy decreases when the velocity increases. This situation is emphasized for *V* = 216 km h^−1^ slightly above the phase speed with a jump between the front and rear side of the tyre.

Figures 5 and 6 give similar results for the one-third octave band 2 kHz. At this frequency, the tyre vibration is highly attenuated so that it propagates like in an infinite space. The vibration is dominated by a free-field regime [16]. The phase speed is *c*_p_ = 131 m s^−1^ and the group speed *c*_g_ = 244 m s^−1^. Again the energy is concentrated at the front while it is extended at the rear. Unlike at 200 Hz, the rolling velocities remain low compared with phase and group speeds. There is no substantial variation in the energy peak magnitude since the free-field regime confines the vibrational energy near the excitation point and the relatively slow velocities do not carry out a significant part of the energy.

The noise emission resulting from the vibrational field in the tyre is beyond the scope of this paper. However, one observation should be made based on background knowledge of tyre/road noise emission. Considering the distribution of vibrational energy on the tyre circumference, it would be expected that the noise emission would be more pronounced to the rear than to the front of the tyre. However, this is usually not the case at least in the low- and medium-frequency domain (*f* ≤ 1.5 kHz), where the tyre/road noise emission is mainly due to the radiation of out-of-plane waves in the tyre carcass. On-board noise measurements (reported in [18], for instance) performed with several microphones placed laterally near test tyres according to ISO 11819-2 standard [19] usually show highest noise levels to the front/side than to the rear/side of the tyre/road contact zone up to nearly 1500 Hz. Stronger impact mechanisms at the leading edge than at the trailing edge are put forward in [18] as part of an explanation. Another factor that may attenuate the front/rear asymmetry in vibrational energy regarding the radiated noise in the medium frequency range is the horn effect created by the geometry between the tyre and road surfaces [20]. The source position strongly influences the noise amplification which can reach more than 15 dB around 1.5 kHz when the source is very close to the contact zone and decreases when the distance between the source and the contact zone increases. Therefore, the horn effect is likely to have more pronounced relative influence on the vibrational energy that is concentrated at the leading edge than that which is driven to the rear at the trailing edge.

## 7. Conclusion

In conclusion, under the assumption of dominant radial vibration, the rolling of thin-walled cylinders on rough surfaces is mathematically equivalent to a periodic, orthotropic and prestressed plate resting on a Winkler foundation and excited by an out-of-plane moving force. The plate model is sufficiently large to include bending and membrane effects as special cases as well as inflation pressure, radius of curvature and centrifugal effects. The closed-form solution presented in this paper allows one to estimate the vibrational energy density in both harmonic and random excitations. Applied to the case of tyre/road contact, the model shows a strong transportation of vibrational energy to the rear and a narrowing of isovalue lines of energy in the front.

## Data accessibility

This article has no additional data.

## Appendix A. Appendix A

This appendix gives the calculation of the integrals *I*_*ij*_(*x*) and *J*_*ij*_(*x*) of equations (5.10) and (5.12). Expanding the product of sine and cosine functions in equations (5.10) and (5.12) gives

A 1 $$\begin{array}{rl} I_{ij}(x,\omega ) & =\frac{1}{2}\int_{0}^{\infty }e^{-\zeta_{ij}\omega_{ij}\tau }\sin\left(\omega_{ij}^{\prime }\tau +2i\pi \frac{x+V\tau }{a}\right)e^{-ı\omega \tau }\;d\tau \\ & \;+\frac{1}{2}\int_{0}^{\infty }e^{-\zeta_{ij}\omega_{ij}\tau }\sin\left(\omega_{ij}^{\prime }\tau -2i\pi \frac{x+V\tau }{a}\right)e^{-ı\omega \tau }\;d\tau \end{array}$$

and

A 2 $$\begin{array}{rl} J_{ij}(x,\omega ) & =\frac{1}{2}\int_{0}^{\infty }e^{-\zeta_{ij}\omega_{ij}\tau }\cos\left(\omega_{ij}^{\prime }\tau +2i\pi \frac{x+V\tau }{a}\right)e^{-ı\omega \tau }\;d\tau \\ & \;+\frac{1}{2}\int_{0}^{\infty }e^{-\zeta_{ij}\omega_{ij}\tau }\cos\left(\omega_{ij}^{\prime }\tau -2i\pi \frac{x+V\tau }{a}\right)e^{-ı\omega \tau }\;d\tau . \end{array}$$

But the Fourier transform of *f*(*τ*) = *H*(*τ*)e^−*ατ*^sin(*βτ* + *φ*) is

A 3 $$\hat{f}(\omega )=\int_{0}^{\infty }f(\tau )e^{-ı\omega \tau }\;d\tau =\frac{\beta \cos\varphi +(ı\omega +\alpha )\sin\varphi }{\beta^{2}+{\left(ı\omega +\alpha \right)}^{2}}$$

and that of *g*(*τ*) = *H*(*τ*)e^−*ατ*^cos(*βτ* + *φ*) is

A 4 $$\hat{g}(\omega )=\int_{0}^{\infty }g(\tau )e^{-ı\omega \tau }\;d\tau =\frac{-\beta \sin\varphi +(ı\omega +\alpha )\cos\varphi }{\beta^{2}+{\left(ı\omega +\alpha \right)}^{2}}.$$

The first integrals in equations (A 1) and (A 2) are obtained by setting *α* = *ζ*_*ij*_*ω*_*ij*_, *β* = *ω*′_*ij*_ + 2*iπV*/*a* and *φ* = 2*iπx*/*a*, while the second integrals require the values *α*, *β*′ = *ω*′_*ij*_ − 2*iπV*/*a*, *φ*′ = − *φ*. This yields

A 5 $$I_{ij}(x,\omega )=\frac{1}{2}\left[\frac{\beta \cos\varphi +(ı\omega +\alpha )\sin\varphi }{\beta^{2}+{\left(ı\omega +\alpha \right)}^{2}}+\frac{\beta^{\prime }\cos\varphi^{\prime }+(ı\omega +\alpha )\sin\varphi^{\prime }}{\beta^{\prime 2}+{\left(ı\omega +\alpha \right)}^{2}}\right]$$

and

A 6 $$J_{ij}(x,\omega )=\frac{1}{2}\left[\frac{-\beta \sin\varphi +(ı\omega +\alpha )\cos\varphi }{\beta^{2}+{\left(ı\omega +\alpha \right)}^{2}}+\frac{-\beta^{\prime }\sin\varphi^{\prime }+(ı\omega +\alpha )\cos\varphi^{\prime }}{\beta^{\prime 2}+{\left(ı\omega +\alpha \right)}^{2}}\right].$$

Since cos*φ* = cos*φ*′ and sin*φ*′ = − sin*φ*, we may re-arrange the terms such that

A 7 $$\begin{array}{r} I_{ij}(x,\omega )=\frac{1}{2}\left[\frac{\beta }{\beta^{2}+{\left(ı\omega +\alpha \right)}^{2}}+\frac{\beta^{\prime }}{\beta^{\prime 2}+{\left(ı\omega +\alpha \right)}^{2}}\right]\cos\varphi +\frac{1}{2}\left[\frac{ı\omega +\alpha }{\beta^{2}+{\left(ı\omega +\alpha \right)}^{2}}-\frac{ı\omega +\alpha }{\beta^{\prime 2}+{\left(ı\omega +\alpha \right)}^{2}}\right]\sin\varphi \end{array}$$

and

A 8 $$\begin{array}{r} J_{ij}(x,\omega )=\frac{1}{2}\left[\frac{ı\omega +\alpha }{\beta^{2}+{\left(ı\omega +\alpha \right)}^{2}}+\frac{ı\omega +\alpha }{\beta^{\prime 2}+{\left(ı\omega +\alpha \right)}^{2}}\right]\cos\varphi +\frac{1}{2}\left[\frac{-\beta }{\beta^{2}+{\left(ı\omega +\alpha \right)}^{2}}+\frac{\beta^{\prime }}{\beta^{\prime 2}+{\left(ı\omega +\alpha \right)}^{2}}\right]\sin\varphi . \end{array}$$

This reduces to

A 9 $$I_{ij}(x,\omega )=\frac{(\beta +\beta^{\prime })(\beta \beta^{\prime }+{\left(ı\omega +\alpha \right)}^{2})\cos\varphi +(ı\omega +\alpha )(\beta^{\prime 2}-\beta^{2})\sin\varphi }{2[\beta^{2}+{\left(ı\omega +\alpha \right)}^{2}][\beta^{\prime 2}+{\left(ı\omega +\alpha \right)}^{2}]}$$

and

A 10 $$J_{ij}(x,\omega )=\frac{(ı\omega +\alpha )(\beta^{2}+\beta^{\prime 2}+2{\left(ı\omega +\alpha \right)}^{2})\cos\varphi +(\beta^{\prime }-\beta )(-\beta \beta^{\prime }+{\left(ı\omega +\alpha \right)}^{2})\sin\varphi }{2[\beta^{2}+{\left(ı\omega +\alpha \right)}^{2}][\beta^{\prime 2}+{\left(ı\omega +\alpha \right)}^{2}]}.$$

Substituting back the values of *α*, *β*, *β*′ and *φ*, we finally get

A 11 $$I_{ij}(x,\omega )=\omega_{ij}^{\prime }\frac{\left[{\left(ı\omega +\zeta_{ij}\omega_{ij}\right)}^{2}+{\omega_{ij}^{\prime }}^{2}-{\left(2i\pi (V/a)\right)}^{2}]\cos2i\pi (x/a)-4i\pi (V/a)(ı\omega +\zeta_{ij}\omega_{ij})\sin2i\pi (x/a\right)}{\left[{\left(\omega_{ij}^{\prime }+2i\pi (V/a)\right)}^{2}+{\left(ı\omega +\zeta_{ij}\omega_{ij}\right)}^{2}][{\left(\omega_{ij}^{\prime }-2i\pi (V/a)\right)}^{2}+{\left(ı\omega +\zeta_{ij}\omega_{ij}\right)}^{2}\right]}$$

and

A 12 $$J_{ij}(x,\omega )=\frac{\begin{array}{c} (ı\omega +\zeta_{ij}\omega_{ij})[{\left(ı\omega +\zeta_{ij}\omega_{ij}\right)}^{2}+{\omega_{ij}^{\prime }}^{2}+{\left(2i\pi (V/a)\right)}^{2}]\cos2i\pi \frac{x}{a}\\ -\;2i\pi (V/a)[{\left(ı\omega +\zeta_{ij}\omega_{ij}\right)}^{2}-{\omega_{ij}^{\prime }}^{2}+{\left(2i\pi (V/a)\right)}^{2}]\sin2i\pi (x/a) \end{array}}{\left[{\left(\omega_{ij}^{\prime }+2i\pi (V/a)\right)}^{2}+{\left(ı\omega +\zeta_{ij}\omega_{ij}\right)}^{2}][{\left(\omega_{ij}^{\prime }-2i\pi (V/a)\right)}^{2}+{\left(ı\omega +\zeta_{ij}\omega_{ij}\right)}^{2}\right]},$$

where $\omega_{ij}^{\prime }=\omega_{ij}\sqrt{1-\zeta_{ij}^{2}}$.
